# Caregivers’ Perspectives of Medication Management Advice for People With Dementia at Hospital Discharge

**DOI:** 10.1093/geroni/igaa057.667

**Published:** 2020-12-16

**Authors:** Mouna Sawan, Yun-Hee Jeon, Christine Bond, Timothy Chen, Sarah Hilmer, Danijela Gnjidic

**Affiliations:** 1 The University of Sydney, Camperdown, Sydney, New South Wales, Australia; 2 University of Aberdeen, Aberdeen, Scotland, United Kingdom; 3 Kolling Institute of Medical Research, St Leonards, New South Wales, Australia

## Abstract

People with dementia admitted to hospitals are more likely to be exposed to inappropriate polypharmacy and experience worse outcomes than people without dementia. Family and informal caregivers play an important role in managing medications across transitions of care; however, studies describing the experiences of medication guidance provided to caregivers at hospital discharge are limited. We have explored caregivers’ perceptions on the quality of and factors that influence caregiver participation in medication guidance at discharge. A qualitative approach using semi-structured interviews was conducted with 29 caregivers of people with dementia across Australia by telephone. Purposive sampling was used to ensure maximum variation of diverse perspectives. Content analysis was used to derive themes. Three themes were derived from analysis: inconsistent approaches to provision of medication information at discharge, caregiver awareness to advocate for the care recipient and managing competing priorities. Some caregivers reported inadequate information was provided because the information was communicated to the patient without the caregiver being present. Other caregivers stated a medication list, discharge summary and discussion with a healthcare profession provided useful information. Caregiver involvement in discussions on medication guidance at discharge was influenced by caregiver awareness to advocate for the care recipient to ensure medication safety and managing competing priorities at the time of discharge to manage stress. Caregivers flagged the need to establish structured caregiver education at discharge and community-based services to manage medications safely. Future studies are needed to explore development of resources to caregiver encourage participation during medication guidance at discharge.

